# Abnormal N400 Semantic Priming Effect May Reflect Psychopathological Processes in Schizophrenia: A Twin Study

**DOI:** 10.1155/2017/7163198

**Published:** 2017-08-28

**Authors:** Anuradha Sharma, Heinrich Sauer, Holger Hill, Claudia Kaufmann, Stephan Bender, Matthias Weisbrod

**Affiliations:** ^1^Research Group Neurocognition, Department of General Psychiatry, Centre for Psychosocial Medicine, Heidelberg University, Heidelberg, Germany; ^2^Department of Psychiatry, University of Jena, Jena, Germany; ^3^Institute of Sports and Sports Science, Karlsruhe Institute of Technology, Karlsruhe, Germany; ^4^Department of General Internal Medicine and Psychosomatics, University of Heidelberg, Heidelberg, Germany; ^5^Department of Child and Adolescent Psychiatry, University Hospital Cologne, Cologne, Germany; ^6^Department of Psychiatry and Psychotherapy, SRH Hospital Karlsbad-Langensteinbach, Karlsbad, Germany

## Abstract

**Objective:**

Activation of semantic networks is indexed by the N400 effect. We used a twin study design to investigate whether N400 effect abnormalities reflect genetic/trait liability or are related to psychopathological processes in schizophrenia.

**Methods:**

We employed robust linear regression to compare N400 and behavioral priming effects across 36 monozygotic twin pairs (6 pairs concordant for schizophrenia/schizoaffective disorder, 11 discordant pairs, and 19 healthy control pairs) performing a lexical decision task. Moreover, we examined the correlation between Brief Psychiatric Rating Scale (BPRS) score and the N400 effect and the influence of medication status on this effect.

**Results:**

Regression yielded a significant main effect of group on the N400 effect only in the direct priming condition (*p* = 0.003). Indirect condition and behavioral priming effect showed no significant effect of group. Planned contrasts with the control group as a reference group revealed that affected concordant twins had significantly reduced N400 effect compared to controls, and discordant affected twins had a statistical trend for reduced N400 effect compared to controls. The unaffected twins did not differ significantly from the controls. There was a trend for correlation between reduced N400 effect and higher BPRS scores, and the N400 effect did not differ significantly between medicated and unmedicated patients.

**Conclusions:**

Reduced N400 effect may reflect disease-specific processes in schizophrenia implicating frontotemporal brain network in schizophrenia pathology.

## 1. Introduction

Language impairment is considered as one of the hallmark symptoms of schizophrenia. Abnormal semantic priming has been reported in schizophrenia patients in various studies that have used different measures and experimental paradigms [1–4]. Semantic priming refers to the facilitated processing of a target stimulus when it is preceded by a semantically related stimulus or context. Traditionally, semantic priming studies in schizophrenia have used reduction in reaction times in primed trials as an index of semantic priming (e.g., [5, 6]). However, more recently, priming studies in schizophrenia have focused on the N400 [7–10] which is a negative-going event-related potential (ERP) peaking at centroparietal scalp sites around 400 ms after the target stimuli that are not primed by the preceding context. N400 was first identified by Kutas and Hillyard as an ERP reflecting semantic association between words elicited 400 ms after the presentation of unexpected endings of sentences [11, 12] and has been researched extensively in the following years (reviewed, e.g., in [13]). Although originally observed in the context of sentences with unpredictable endings, N400 can also be evoked by isolated words, for example, the target word in a lexical decision task, and the amplitude of the N400 is considerably modified by the semantic relatedness of a previously presented word (prime) [13, 14]. Targets that have been primed by the preceding context show a reduced (less negative/more positive) N400 and this reduction, called the N400 effect, has been attributed to the activation of related semantic networks by the prime leading to facilitated processing of the target [15].

Although many studies have found the N400 effect to be abnormal in schizophrenia, the question of whether these abnormalities reflect trait markers of schizophrenia or rather reflect disease-related processes is unclear. Studies that have addressed this issue in a family design [16–19] have used different paradigms to evaluate N400 abnormalities in first-degree family members of schizophrenia patients and have reported inconsistent results. Kimble et al. (2000) [16] found the N400 effect in a sentence paradigm to be reduced in high schizotypy individuals but not in unaffected family members of schizophrenia patients. Kiang et al. (2014) [18] reported no differences between healthy controls and unaffected first-degree relatives of schizophrenia patients for the N400 effect; Guerra et al. (2009) [17], on the other hand, found a reduced N400 effect also in unaffected first-degree family members of patients. Pfeifer et al. (2012) [19] reported an abnormal N400 effect only for the indirect semantic condition in schizophrenia patients compared to healthy controls but failed to find differences between unaffected siblings and controls. Two studies looking at effects of medication have reported only limited effects of medication on the N400 amplitude during word recognition [20] and N400 priming effect in a lexical decision task [21] in schizophrenia patients. A useful alternative approach to examine this issue is to compare monozygotic twins concordant and discordant for schizophrenia with healthy twin pairs. Unlike first-degree relatives (who share only 50% of their genetic material), monozygotic twins share 100% of their genes and differences in phenotypic traits can be attributed to differential environmental exposure and/or disease or epigenetic processes. Although different more complex models may be employed to address the question of genetic versus environmental processes, the twin study design provides enough theoretical grounding and power for qualitative modeling of these effects and has been employed widely across heritability studies (e.g., [22]). We compared the N400 effect across a group of monozygotic twin pairs concordant and discordant for schizophrenia/schizoaffective disorder and healthy control pairs as they performed a lexical decision task (other data from the same subject sample has been included in previous studies [23–26]). To our knowledge, this is the first study to look at the N400 effect in schizophrenia in a twin design. We speculated that this approach may shed light on whether abnormal N400 effect in schizophrenia reflects trait liability to schizophrenia and therefore can be a potential endophenotype, or is more related to the clinical state and therefore can be established as a potential marker for psychopathological processes in schizophrenia.

## 2. Materials and Methods

### 2.1. Subjects

36 monozygotic twin pairs entered the study: 6 concordant pairs (*N* = 12 for the concordant affected group) with both twins affected by schizophrenia/schizoaffective disorder, 11 discordant pairs where only one twin was affected by schizophrenia/schizoaffective disorder (therefore, *N* = 11 for each of the affected and unaffected discordant groups), and 19 concordant control pairs (*N* = 38 for the concordant healthy group) where both twins were psychiatrically healthy. Affected twins were identified from the records of different psychiatric departments in and around Heidelberg. ICD-10 diagnoses were established in all subjects by the Schedules for Clinical Assessment in Neuropsychiatry (SCAN) [27]. Affected twins fulfilled the criteria for a diagnosis of the F2 (schizophrenia, schizotypal and delusional disorders) category of ICD-10. Schizoaffective and schizotypal disorders were included since they are considered a part of the schizophrenic genotype and the highest heritability quotients have been reported when these diagnoses are included as a schizophrenic phenotype in twin studies [28, 29]. Similar results have been reported for family/adoption studies showing a genetic relationship between these diagnoses [30]. The unaffected discordant cotwins did not have any of the F2 diagnoses. However, two of the cotwins fulfilled the criteria F32.10 for moderate depressive episode without somatic symptoms and one fulfilled the criteria F31.7 for remitted bipolar disorder. Current psychopathological status was assessed using the Brief Psychiatric Rating Scale (BPRS) [31]. Handedness was measured using the Edinburgh Handedness Inventory [32]. Clinical characteristics of the subject population are given in Table 1.

At the time of the recordings, 13 patients (5 discordant affected and 8 concordant affected) were receiving antipsychotic medications with a mean dose equivalent to 469 mg/day of chlorpromazine. In addition, 3 affected twins were taking antidepressants at the time of the study. One discordant nonschizophrenic twin was taking lithium. Controls were recruited through newspaper advertisements in the Heidelberg area. They had no personal or family history of mental illness, based on the Family History Research Diagnostic Criteria. None of the subjects had a history of neurological disorder or head injury. Substance abuse history was measured using the SCAN and all subjects that were using drugs or* Cannabis* at the time of investigation or had a history of long-term drug abuse were excluded from the study. Zygosity was diagnosed by DNA microsatellite analysis [33]. After a complete description of the study, subjects provided written informed consent and were paid for participation.

### 2.2. Stimuli and Task

In the lexical decision task, subjects were presented with stimuli consisting of 216 pairs of letter strings (primes and targets). While primes were always German words, 50% of the targets were real German words and the other 50% were legally spelled pseudowords. The 108 prime-target combinations involved real words as targets involving 36 nonrelated pairs, 36 indirectly related pairs, and 36 directly related pairs. Examples of primes and targets and mean values for word frequency class and length for each condition are provided in Table 2.

Further details of the stimulus material and word selection are provided in a previous study [34]. The word frequencies were determined according to [35] and the Leipzig Online Dictionary (http://wortschatz.uni-leipzig.de/). The primes as well as targets did not differ across conditions for word length and frequency class (one-way ANOVA with condition as a factor was nonsignificant for primes as well as target words, *p* > 0.05).

**Figure 1:**
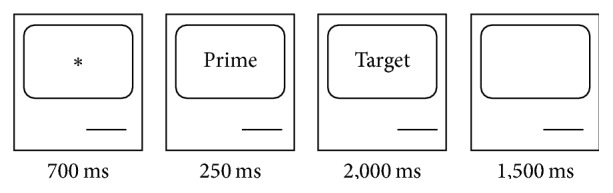
The lexical decision task. Prior to the trial, the computer screen was blank. A trial started with a fixation point (*∗*) presented for 700 ms followed by presentation of the prime for 250 ms which was immediately succeeded by the presentation of the target word. The target was displayed for 2 s, after which the screen went blank for 1.5 s.

Subjects were seated 60 cm from the monitor in an electrically shielded dimly lit room. The word stimuli subtended a visual angle of about 1 to 2 degrees in width and 0.5 degrees in height on the monitor (Stim, Neuroscan Inc., El Paso, USA). The prime was presented for 250 ms followed by the target word (word or a pseudoword) presented for 2 s. Subjects had to read the words and respond as quickly and accurately as possible to whether the target word was a real German word or not by pressing the left mouse button with the index finger of their dominant hand (real words) or the right mouse button with the middle finger of the dominant hand (pseudowords). Trials were presented continuously (stimulus onset asynchrony (SOA), 250 ms between the prime and the target, intertrial interval of 1.5 s). Before the start of every trial, a fixation point was presented for 700 ms. Sequence of one trial is displayed in Figure 1. The trials were presented in two blocks. A break of up to 5 minutes was allowed between the presentation blocks. Subjects practiced with 10 trials before the start of the main test.

### 2.3. Electroencephalography (EEG) Data

**Figure 2:**
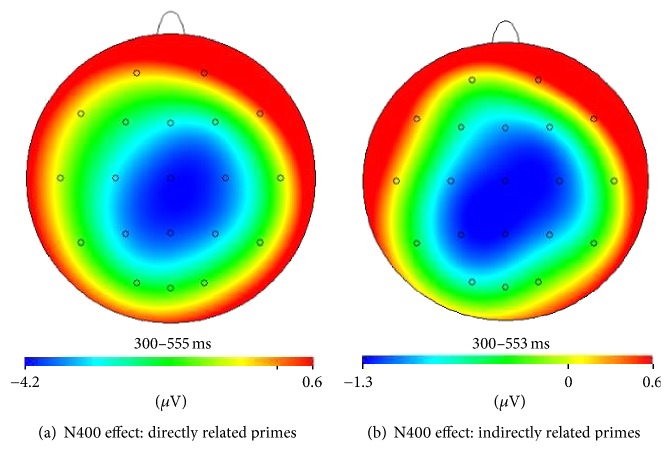
Topographical maps depicting the distribution of (a) direct and (b) indirect N400 priming effect (in microvolts) across the scalp for the concordant healthy control group. The effect was centered around the medial electrode site (Cz).

EEG was continuously recorded (low-pass filter 70 Hz, A/D rate 400 Hz) with a SynAmps DC amplifier using the software Scan 3.0 (Neuroscan Inc., El Paso, USA) from 20 sintered Ag/AgCl electrodes positioned according to the international 10–20 system. Linked mastoids were used as reference and FPz was used as ground. Vertical EOG was recorded with supra- and infraorbital electrodes. Electrodes on the external canthi recorded horizontal EOG. Electrode impedance was maintained below 5 k*Ω* for all recordings. Continuous EEG was segmented offline in 1400 ms epochs (−400 to 1000 ms, target word onset at 0 ms) and a low-pass filter of 16 Hz (24 dB/octave) was applied. Correction for ocular artefacts was performed using regression-based weighting coefficients [36]. Before baseline correction, signals outside the amplifier range were removed. After baseline correction, a semiautomatic artefact criterion was applied as follows: amplitude criterion ±50 *μ*V and gradient criterion 10 *μ*V/segment. To monitor the procedures for artefact rejection and correction of eye blinks, the complete datasets were inspected visually and obvious artefacts surviving these thresholds were removed as well. On the other hand, when typical (physiological) EEG activity was larger than these thresholds, the relevant segments were reincluded. Furthermore, only epochs in which subjects responded correctly between 200 and 2000 ms after the target word were included in further analysis. The average number of trials included in the study for each condition was 28 for nonrelated condition, 30 for indirectly related condition, and 29 for directly related condition. All subjects had 18 minimum trials for every condition except two subjects (belonging to concordant schizophrenia group) who had an average of 13 and 8 trials. ERPs for all subjects were calculated by averaging the trials time locked to the target word, relative to a baseline of −400 to −300 ms. N400 amplitude was measured as the mean voltage between 300 and 550 ms after stimulus at the Cz electrode separately for the three prime-word conditions. The use of Cz electrode is in agreement with the reported centroparietal distribution of the N400 priming effect in lexical decision tasks (e.g., [8, 9, 37]) and with the topographical scalp distribution obtained for our data where the N400 effect was maximal at centroparietal sites for both the direct and the indirect conditions in healthy controls (Figure 2). The ERP analysis was carried out using the Scan 3.0 software (Neuroscan Inc., El Paso, USA).

### 2.4. Statistical Analysis

Statistical analysis was performed using Stata 8 (StataCorp, College Station, Texas, USA). The “survey” option in Stata allows for nonindependent observations and takes into account the similarity between twins of a pair by calculating a robust sandwich estimator to estimate standard errors. The relatedness between twins of a pair (cluster correlation) violates the independent observations assumption in the analysis of variance, and the sandwich estimator corrects for this bias providing robust estimates of 95% confidence intervals, standard errors, and *p* values [38–41]. All the performed analyses reported below were performed using the “survey” option in Stata and take into account the nonindependence of observations from twins belonging to the same pair.

The twin groups were compared on various measures (reaction time priming effect, mean N400 amplitude, and N400 priming effect) using a multiple regression model which included group (concordant affected, discordant affected, discordant unaffected, and concordant healthy) as the predictor variable and age and gender as covariates (wherever specified in the Results). The variable “group” which is a 4-level categorical variable was coded into 3 dummy variables (each reflecting the presence or absence of a particular group with the healthy concordant group as the reference group). This recoding facilitated the calculation of individual contrasts after regression. In order to test for the collective effect of the factor group, Wald test was used. When the main effect of group reached significance, planned comparisons were made to compare the concordant affected, discordant affected, and discordant unaffected groups with the healthy concordant group.

To further examine whether the measures yielding significant group differences were correlated with the clinical state of the patients, we carried out an exploratory correlation analysis using regression between the relevant measures that yielded group differences in the aforeementioned analysis as the predictor variable and total BPRS scores in the affected twins (both concordant and discordant affected taken together) as the dependent variable (age and gender as covariates). BPRS evaluates the psychopathological status of the patients [31] and therefore a significant correlation with BPRS score would implicate the marker as reflecting disease-specific processes.

To verify whether the obtained pattern of results was due to medication effects, we compared the patients that were taking antipsychotic medication with those that were not taking anitpsychotic medication at the time of the study on relevant measures using a* t*-test.

To rule out the effects of attention on the processing of primes, we compared across groups the amplitude of a visual evoked component P1, a positive deflection that occurs around 100 ms after stimulus presentation at posterior electrodes and is thought to reflect sensory processing in the ventral visual stream and is modulated by attention [42, 43]. For the purpose, we analyzed and compared mean voltage values between 100 ms and 175 ms after prime onset at the Pz electrode across the groups for the nonrelated condition.

## 3. Results

### 3.1. Behavioral Measures

Groups did not differ significantly in the number of correct responses (main effect of group: *p* > 0.3 for all three conditions) although the concordant affected group tended to make more errors than the discordant or the healthy control groups. Mean reaction times (RTs) for the correct trials were calculated for all subjects and conditions. Trials with RTs exceeding twice the mean reaction time for all trials were excluded from the calculation. RT priming effects were calculated by subtracting mean RTs in the directly related condition and the indirectly related condition from the nonrelated condition, yielding direct and indirect RT priming effects, respectively. Multiple regression (after controlling for age and gender) yielded no significant effect of group on both the indirect (*p* = 0.9) and the direct (*p* = 0.6) RT priming effects. RT priming effects for the four groups are given in Table 3. As seen from mean values in Table 3, the direct RT priming effect was larger than the indirect RT priming effect in all groups, which was expected since the time taken to react to words preceded by directly related primes would be smaller (facilitation via prime) than to those preceded by indirectly related primes.

### 3.2. Electrophysiological Measures

**Figure 3:**
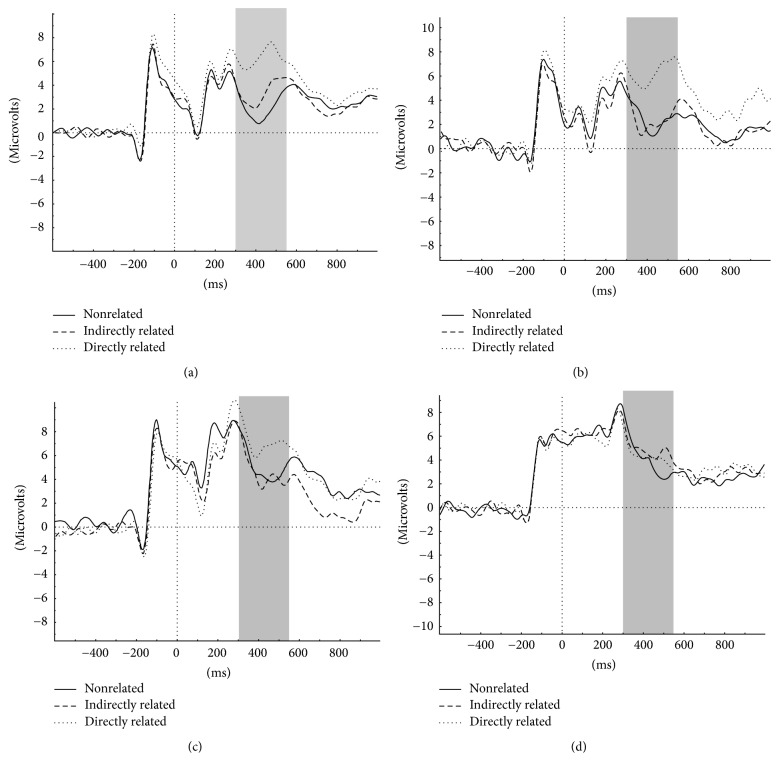
Grand-averaged EEG waveforms at the Cz electrode depicting the course of the ERPs across the three conditions and the four monozygotic twin groups: (a) concordant healthy, (b) discordant unaffected, (c) discordant affected, and (d) concordant affected.

**Figure 4:**
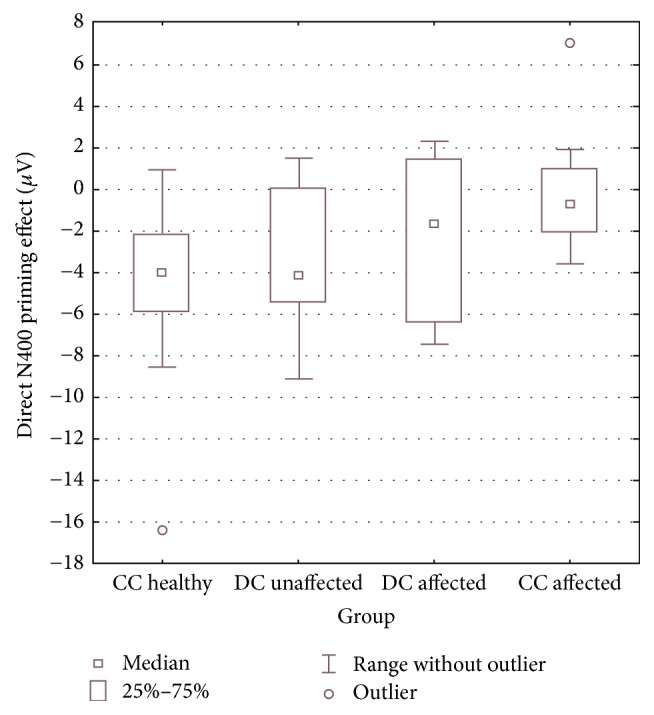
Box plot depicting the distribution of direct N400 priming effect (in microvolts) across the monozygotic twins: concordant healthy (CC healthy, *N* = 38), discordant unaffected (DC unaffected, *N* = 11), discordant affected (DC affected, *N* = 11), and concordant for schizophrenia/schizoaffective disorder (CC affected, *N* = 12).

N400 priming effect was calculated by subtracting N400 amplitudes in the directly related condition and the indirectly related condition from that in the nonrelated condition, yielding direct and indirect N400 priming effects, respectively. Multiple regression (after controlling for age and gender) yielded a significant main effect of group only for the direct priming effect (*F*(3, 33) = 9.5, *p* = 0.003). The effect of group for the indirect priming effect was not significant (*F*(3, 33) = 1.1, *p* = 0.4). Planned contrasts with respect to the healthy control group for the direct priming effect revealed that the concordant affected twins exhibited significantly reduced (less negative) direct priming effect as compared to the control twins (*t* = 4.0, *p* < 0.001). Also the discordant affected twins showed a statistical trend for having a lower direct priming effect than the control group (*t* = 1.9, *p* = 0.07). The discordant unaffected and the healthy control twins did not differ significantly from each other (*t* = 0.52, *p* = 0.6). We employed one further exploratory regression to compare the discordant affected and the discordant unaffected group on the direct N400 priming effect. However, the comparison failed to reach significance (*p* > 0.3). Groups did not differ significantly (after controlling for age and gender) in the mean N400 amplitude (main effect of group: *p* > 0.2 for all three conditions), although the affected twins showed lower (less negative) mean values in the nonrelated and indirectly related conditions. Mean N400 amplitudes and N400 priming effects for the four groups are given in Table 3. The ERP waveforms for the three conditions for all groups are shown in Figure 3 and the distribution of the direct N400 priming effect for all groups is shown in Figure 4.

### 3.3. Correlation with BPRS

After controlling for the effects of age and gender, BPRS and direct N400 priming effect showed a statistical trend for significant positive correlation in the affected twins (standardized beta = 0.4, *p* = 0.07); lower (less negative/more positive) semantic priming effect was associated with higher BPRS score, which in turn indicated that lower direct N400 priming effect in concordant and discordant ill twins could be likely associated with disease-related processes. Mean BPRS scores are given in Table 1.

### 3.4. Effect of Medication

A comparison of the direct N400 effect in affected twins taking antipsychotic medication with those not on antipsychotic medication revealed no significant differences (*t* = 1.05, *p* = 0.3). This indicated that the pattern of group differences for the direct N400 effect was not affected by medication status.

### 3.5. Effect of Attention on Prime Processing

The four groups did not differ on the post-prime P1 amplitudes for the nonrelated condition, the main effect of group being nonsignificant (*F*(3, 33) = 0.16, *p* = 0.91), indicating that the primes were not differentially attended to and processed in the four groups.

## 4. Discussion

This study compared N400 semantic priming effects across a sample of monozygotic twins concordant and discordant for schizophrenia and healthy monozygotic twins and found significant differences between the groups only on the N400 priming effect for the direct condition. The effect of group on mean N400 amplitudes on the other hand failed to reach significance. These results show that N400 priming effect reflecting the ease of spread of activation in semantic networks may be a more sensitive measure of semantic network disturbances in schizophrenia as compared to the mean N400 amplitude that reflects the absolute strength of semantic processing. For the direct N400 priming effect, twins concordant for schizophrenia had a significantly lower N400 effect as compared to the healthy control twin pairs. In addition, the discordant affected twins showed a trend for having a lower direct N400 effect as compared to the control twins. The discordant unaffected twins, however, did not differ significantly from the control twins. Given that monozygotic twins share 100% of their genetic material, the N400 effect reduction observed in the given task only in the affected twins indicates that this reduction may reflect disease-specific processes in schizophrenia and not so much trait liability, making it a potential candidate marker for detecting and elucidating psychopathological mechanisms in schizophrenia. This was further verified by the trend for significant correlation between direct N400 priming effect and BPRS score in the affected twins, with lower priming effect associated with higher BPRS scores. Since the number of correct responses and the post-prime P1 amplitudes did not differ significantly across the groups, this ruled out noncompliance with the task or generalized attention deficit as a reason for the obtained group differences in the N400 effect.

The result that the discordant affected and unaffected twins showed no significant differences for the direct N400 priming effect, even when the discordant affected twins showed lower mean values for this effect (Table 3), could have been due to a lack of power as the group size was quite small (*N* = 11 discordant pairs). An examination of this effect in a bigger sample would be necessary to verify this result. Also, that we obtained only a statistical trend for the correlation between BPRS score and direct N400 priming effect could be due to a lack of power and to the fact that the global BPRS score is only a very broad measure of psychopathology in schizophrenia. More specific measures assessing clinical status would have to be employed to elucidate the precise relationship between the N400 priming effect and schizophrenia symptomatology.

That the priming deficits in patients were only evident for the N400 effect and not for the RT effect provided evidence that the N400 effect may be a more sensitive marker for detecting semantic priming deficits in schizophrenia as compared to behavioral priming measures.

To our knowledge, this is the first study that has looked at the trait versus state validity of N400 effect abnormalities in schizophrenia using a twin design. Amongst the studies that have investigated this question using a family design [16–19], our results are consistent with the results of Kimble et al. (2000) [16] who used a sentence paradigm and found the N400 effect to be reduced in high schizotypy individuals but not in unaffected family members of schizophrenia patients. Even though the sentence paradigm used in their study reflects contextual processes as compared to our paradigm which reflects spreading of activation in semantic networks, their results are in agreement with a similar indication from our results (where both concordant and discordant affected twins showed lower N400 effect than the healthy control twins while the discordant unaffected twins were not significantly different from the control twins). Although Guerra et al. (2009) [17] reported different findings, where they found a reduced N400 effect also in unaffected first-degree family members of patients, there could be several reasons for this. Firstly, their task was very different from the present study where they used a picture matching task involving explicit semantic matching of stimuli as opposed to the lexical decision task (where semantic priming is implicit) used in the present study. Secondly, the stimulus onset synchrony (SOA) between the two consecutive pictures in their study was 1200 ms as opposed to the 250 ms SOA in the present study. Explicit semantic matching and a long SOA together would elicit many more strategic and top-down control processes [44] as compared to the task in the present study which involved some control processes (in addition to the spreading of activation in semantic networks), but not to the same degree as in Guerra et al.'s study. Our results are also consistent with Kiang et al. (2014) [18], who reported no significant differences between healthy controls and unaffected first-degree relatives of schizophrenia patients for the N400 effect during a lexical decision task across SOAs, and with Pfeifer et al. (2012) [19], who reported a similar lack of difference between unaffected siblings and controls. Another case report compared two twins of a pair discordant for schizophrenia on N400 repetition effects [45]. They reported reduced N400 repetition effects for both twins of the pair, but again the repetition task can be seen as an extreme case of a long SOA where repeated words were separated by long time intervals and long-term memory processes would be involved. These differences make it hard to compare the results of these studies and further work will be required to resolve the significance of these differences. They further highlight the fact that the significance of the N400 effect depends on the task conditions employed and should be interpreted accordingly. N400 effect derived from another task using different conditions (e.g., longer SOAs) could involve different (e.g., more control) processes and hence may reflect other aspects of schizophrenia pathology.

In general, our results are also in consensus with recent studies which have shown a reduced N400 effect in schizophrenia patients in tasks involving lexical decision-making [4, 8, 9]. Our results also make sense in terms of the distributed network model of the N400 effect. N400 effect is thought to reflect the activity of the frontotemporal semantic networks [15, 46–48]. Abnormal functional connectivity in frontotemporal networks has been implicated previously in schizophrenia [49–51] and reduced N400 priming effect may reflect this altered connectivity [52]. We are aware of only one study that has examined the trait liability component of frontotemporal connectivity in schizophrenia [53] and although this study found abnormal frontotemporal connectivity in siblings of schizophrenia patients as compared to healthy controls, the trait validity of connectivity measures was only found to be indirect, with low heritability and relative risk values. Another study [52] using event-related fMRI reported reduced frontotemporal connectivity during a semantic decision task in schizophrenia patients with formal thought disorder. Further verification of the connectivity hypothesis would require direct analysis of frontotemporal coherence in EEG data.

We found no differences in the direct N400 effect between medicated and unmedicated patients. This is in line with previous evidence which has shown reduced N400 effect in unmedicated patients with schizophrenia [54] and is consistent with another study showing limited effects of medication on N400 priming effect in schizophrenia patients [21].

One point to be noted is the presence of outliers in the concordant healthy and concordant affected groups (Figure 3). We ran the same analysis after excluding the outliers and the pattern of group differences for the direct N400 priming effect remained unchanged (main effect of group, *F*(3, 33) = 10.9, *p* < 0.001). Also, as evident from Figure 2 and Figure 3 and from mean values in Table 3, the concordant affected twins showed a larger reduction of the direct N400 priming effect compared to discordant affected twins. This could indicate some genetic effects on N400 priming deficits in line with the proposition that concordant affected twins may carry more genetic liability to schizophrenia than discordant affected twins [24]. Although this dampens the interpretation of N400 priming deficits as indicating environmental effects on schizophrenia, but given that both groups of affected twins showed N400 priming deficits (even if the concordant affected group had lower mean values than the discordant affected group), the interpretation of these deficits as markers of pathophysiology/disease-related processes in schizophrenia still holds (independent of whether pathophysiology arises from genetic or environmental effects). Also, it could point to stronger psychopathological manifestation of schizophrenia in the concordant affected group as also evident from the higher mean BPRS scores in the concordant affected group compared to the discordant affected group. This was furthermore in agreement with the trend for correlation between BPRS score and direct N400 priming effect in the affected twins and may not be surprising as there is some evidence pointing to more severe clinical impairments in monozygotic twins concordant for schizophrenia spectrum disorders [55, 56].

One limitation of the present study was the small sample size which may have led to power constraints. More rigorous test of this hypothesis would need replication in a bigger sample size.

## 5. Conclusions

The results from the present study add important evidence towards the utility of the direct N400 effect during short SOAs as a marker for predominantly environmental/disease-related processes in schizophrenia and implicate disturbed connectivity of frontotemporal networks in schizophrenia psychopathology. This could have important implications for elucidating pathophysiological mechanisms and developing relevant clinical markers for diagnosis and treatment of schizophrenia.

## Figures and Tables

**Table 1:** Clinical characteristics: mean values (standard deviation).

Group	*N*	SCAN	Age [years]	Gender [M: male; F: female]	Handedness [R: right; L: left; AMBI: ambidextrous]	Schooling [years]	BPRS
Concordant affected (both twins of the pair diagnosed with schizophrenia/schizoaffective disorder)	12	12 schizophrenia	31.2 (7.6)	4 F, 8 M	11 R, 1 L	11.1 (2.4)	35.2 (17.3)

Discordant affected (twins of discordant pairs diagnosed with schizophrenia/schizoaffective disorder)	11	7 schizophrenia 1 schizotypal 3 schizoaffective	31.1 (10.5)	5 F, 6 M	11 R	10.5 (1.4)	28.5 (7.4)

Discordant unaffected (twins of discordant pairs not diagnosed with schizophrenia/schizoaffective disorder)	11	—	31.1 (10.5)	5 F, 6 M	8 R, 2 L, 1 AMBI	10.9 (1.8)	—

Concordant healthy (both twins of the pair healthy)	38	—	32.1 (10.2)	20 F, 18 M	36 R, 1 L, 1 AMBI	11.3 (2.0)	—

SCAN: Schedules for Clinical Assessment in Neuropsychiatry; BPRS: Brief Psychiatric Rating Scale.

**Table 2:** Types and number of conditions (prime-target relations) used in the experiment.

Condition	*N*	Prime examples	Frequency class (mean ± SE)	Length (mean ± SE)	Target examples	Frequency class (mean ± SE)	Length (mean ± SE)
Directly related	36	Bein (Leg)	66.5 ± 21.0	5.1 ± 0.2	Arm (Arm)	67.5 ± 21.1	5.1 ± 0.2
Indirectly related	36	*Löwe* (Lion)	62.0 ± 25.3	5.0 ± 0.2	*Streifen* (Stripes)	32.3 ± 11.5	5.4 ± 0.2
Nonrelated	36	*Anker* (Anchor)	32.3 ± 11.0	5.5 ± 0.3	*Herr *(Mister)	22.3 ± 7.1	5.1 ± 0.2

*Note*. SE: standard errors.

**Table 3:** Behavioral and EEG measures: mean values (standard deviation).

Measure	Concordant affected	Discordant affected	Discordant unaffected	Concordant healthy
*Number of correct responses*				
Nonrelated	34.6 (1.3)	35.2 (1.4)	35.1 (1.0)	35.1 (1.5)
Indirectly related	34.6 (2.2)	35.6 (0.7)	35.7 (0.5)	35.6 (1.2)
Directly related	34.8 (1.9)	35.8 (0.4)	35.6 (0.7)	35.8 (0.7)
*Reaction time priming effect (ms)*				
Directly related	122.0 (81.3)	124.0 (69.4)	126.6 (75.0)	120.9 (42.3)
Indirectly related	84.3 (49.7)	59.5 (42.2)	52.5 (49.7)	68.1 (42.4)
*N400 amplitudes (μV)*				
Nonrelated	4.1 (3.9)	5.1 (6.9)	2.4 (6.2)	2.1 (7.3)
Indirectly related	4.9 (5.2)	4.6 (6.6)	2.7 (5.8)	3.4 (5.4)
Directly related	4.3 (3.2)	7.1 (6.4)	6.2 (6.2)	6.3 (5.9)
*N400 priming effect (μV)*				
Directly related	−0.2 (2.8)	−2.1 (3.8)	−3.7 (3.5)	−4.3 (3.1)
Indirectly related	−0.7 (2.6)	0.4 (2.7)	−0.3 (1.2)	−1.3 (3.5)
